# Efficient Continuous Skyline Query Processing in Wireless Sensor Networks

**DOI:** 10.3390/s19132902

**Published:** 2019-06-30

**Authors:** Yingyuan Xiao, Xu Jiao, Hongya Wang, Ching-Hsien Hsu, Li Liu, Wenguang Zheng

**Affiliations:** 1 Key Laboratory of Computer Vision and System, Ministry of Education, Tianjin University of Technology, Tianjin 300384, China; 2 Tianjin Key Laboratory of Intelligence Computing and Novel Software Technology, Tianjin University of Technology, Tianjin 300384, China; 3 College of Computer Science and Technology, Donghua University, Shanghai 201620, China; 4 Department of Computer Science and Information Engineering, Asia University, Taichung 41354, Taiwan

**Keywords:** wireless sensor networks, skyline query, continuous skyline query

## Abstract

Owing to the rapid advent of wireless technology and proliferation of smart sensors, wireless sensor networks (WSNs) have been widely used to monitor and query the physical world in many applications based on the Internet of Things (IoT), such as environmental monitoring and event surveillance. A WSN can be treated as a distributed database to respond to user queries. Skyline query, as one of the popular queries for multi-criteria decision making, has received considerable attention due to its numerous applications. In this paper, we study how to process a continuous skyline query over a sensor data stream in WSNs. We present an energy-efficient continuous skyline query method called EECS. EECS can avoid the transmission of invalid sensor data and prolong the lifetime of WSNs. Extensive experiments are conducted, and the experimental results demonstrate the effectiveness of the proposed method.

## 1. Introduction

As the development of wireless technology and proliferation of smart sensors, wireless sensor networks (WSNs) become important data sources and have been widely used in many applications [1,2,3]. A WSN normally consists of a large number of distributed sensor nodes that organize themselves into a multi-hop wireless network. WSNs can provide continuous sensor data stream for a variety of applications, and the challenge is how to extract the valuable information from the sensor data stream for these applications because of the limited battery resource on each sensor node. A WSN can be treated as a distributed database to respond to user queries. TinyDB [4] and Cougar [5] are two kinds of typical sensing data query system based on WSNs. However, limited by the performance of the hardware, they can only carry out some basic operations, such as MAX, MIN and other simple aggregation queries. With the improvement of the WSNs’ hardware performance, the researchers have begun to study some complex queries, such as Top-k, join, Skyline and so on.

Skyline query, as one of the popular queries for multi-criteria decision making, provides an efficient method for extracting the valuable information from multi-dimensional datasets. Given a multi-dimensional data set *P* containing data *p*_1_, *p*_2_, ..., *p_n_*, the skyline query over *P* retrieves the set of all *p_i_* in *P* such that no $p_{j}$ dominates $p_{i}$. We say $p_{j}$ dominates $p_{i}$, if $p_{j}$ is better than $p_{i}$ with respect to at least one attribute, and is at least as good as $p_{i}$ on all other attributes. Most of the existing skyline query algorithms assume a static dataset, where data is often kept relatively stable unless an explicit update operation occurs. However, data is generated in real-time and dynamically changes over time in many practical applications. For example, the sensor data captured by sensor nodes is dynamic over time in WSNs. Therefore, the skyline query in WSNs must process query over a sensor data stream, which is different from skyline query processing on a static dataset. This kind of skyline query over dynamic data stream can be grouped into two categories, i.e., snapshot skyline query and continuous skyline query. The snapshot skyline query over a data stream is similar to the traditional skyline query on a static dataset. That is, it first gets a static snapshot of the data stream at the current time and then retrieves those data objects not dominated by others on the static snapshot. In contrast, the continuous skyline query over a data stream involves a dynamic dataset where data objects are continually being added or removed according to their arrival time and expiration time. Obviously, the continuous skyline query over a data stream is more complicated than the snapshot skyline query, and the existing skyline query algorithms based on a static dataset are not suitable for processing the continuous skyline query over a sensor data stream in WSNs. In addition, most sensor nodes in a WSN are battery powered, so power consumption should be minimized when processing query tasks. The energy cost in the query processing procedure consists of the communication cost and the computation cost of the sensor nodes because the communication cost for transmitting one bit by radio is typically no less than the computation cost for executing 1,000 CPU instructions [6]. We can consider the communication cost as the energy cost when the time complexity of the algorithm running on each sensor node is relatively low, i.e., linear to the data size. Since the energy overhead on communication dominates the total energy consumption of a sensor [7], the challenge for the continuous skyline query in WSNs is to fulfill the query task with minimum communication cost. In this paper, we address the problem of continuous skyline query processing in WSNs and present an energy-efficient continuous skyline query method. 

The rest of this paper is organized as follows. Section 2 reviews related work on skylines queries. Section 3 defines the problem studied in this work. Section 4 presents the processing method for continuously computing the current skyline query result over a sensor data stream in WSNs. Section 5 evaluates our method through extensive experiments, and Section 6 concludes this paper.

## 2. Related Work

The skyline query was first introduced into the data management community by Borzsonyi et al. [8]. Since then many skyline query algorithms over a static data set have been proposed for conventional centralized environment [9,10,11,12,13]. 

However, for many emerging streaming applications, such as intelligent transport, real time monitoring, etc., the values of data objects are dynamically changing as the states of the monitored objects are updated. The conventional skyline query algorithms over a static data set have been difficult to adapt to the dynamic data stream. Some continuous skyline query algorithms have been proposed for dynamic data streams [14,15,16]. Tao et al. study skyline query in a stream environment, where query processing takes into account only a “sliding window” covering the most recent data objects and propose algorithms that continuously monitor the incoming data and maintain the skyline incrementally [14]. Wu et al. address the problem of efficient maintenance of a materialized skyline view in response to skyline removals [15]. Hsueh et al. explore the problem of maintaining continuous skyline queries efficiently over dynamic objects with *d* dimensions and propose an efficient update approach for skyline computations, which facilitates an efficient and incremental skyline update strategy to create a pre-computed second skyline set [16]. He et al. propose a multiple layer grids scheme for efficiently processing continuous skyline queries over skewed data set. 

The abovementioned continuous skyline query algorithms are all for a centralized computing environment and inapplicable to WSNs, where the data stream is produced by a large number of distributed sensor nodes. In order to reduce the energy cost caused by the data transmission between nodes in WSNs, most of the skyline query algorithms in WSNs are based on the pruning strategy [17,18]. Wang et al. [17] propose an energy-efficient skyline query method for multidimensional sensing data. The method uses a node cut strategy to dynamically generate filtering tuples when collecting query results instead of issuing queries with filters. Roh et al. [18] propose a filter-based method for two-dimensional skyline query processing in WSNs, which provides an enhanced efficiency by reduction of the total wireless communication between sensor nodes. Furthermore, more aspects of skyline computation in WSNs, such as G-skyline query [19] and Geometry-Based Distributed Spatial Skyline Query [20], have been studied. However, none of these methods described above provides support for continuous skyline queries. Chen et al. [21] propose a MINMAX approach for continuous skyline query in WSNs. The MINMAX approach utilizes properties of the MinMax operator to maintain the hierarchical threshold and to promote efficiency of skyline computation. The disadvantage of this method is that the root node is required to send the local result set to leaf nodes, and the leaf nodes also need to report the updated result set when the skyline result is updated every time. Xin et al. [22] present an energy efficient algorithm i.e. SWSMA. SWSMA only calculates the data in the sliding window, which is the latest data for skyline query, and two filtering strategies are adopted, namely tuple filtering and grid filtering to reduce the amount of data forwarding.

## 3. Preliminary

Let *S* be the set of data objects perceived by sensor nodes in a sensor network. Each data object *d* in *S* has a set of attributes denoted by *A* = {*a*_1_, *a*_2_, …, *a_m_*}. We use *d*[*a_i_*] to denote the *i*-th attribute value of *d*, which represents a certain feature of a perceived external object, such as temperature, humidity, and so on. In addition, unlike static data objects, each data object *d* in *S* has an arrival time and an expiration time associated with it. Let *t_arr_*(*d*) and *t_exp_*(*d*) represent the arrival time and the expiration time of *d*, respectively, which defines *d* as valid in the time interval [*t_arr_*(*d*), *t_exp_*(*d*)]. We use *S_c_* to denote the current valid snapshot of *S*, which contains all currently valid data objects of *S*. Suppose *t_c_* represents the current time, then *S_c_* = {*d*|*t_arr_*(*d*)$\;\leq t_{c}\leq \;$*t_exp_*(*d*) and *d* ∈ *S*}.

In the following, we formally define continuous skyline query and related concepts.

**Definition** **1.** **Data object**. *A data object d in S is defined as a multi-tuple, i.e., d = (id, d[a_1_], d[a_2_], …, d[a_m_], t_arr_(d), t_exp_(d)), where id is the unique identifier for each data object.*

**Definition** **2.** **Dominance**. *Given two data objects d_j_ and d_k_, we say d_j_ dominates d_k_ denoted by d_j_≺ d_k_, if d_j_ is better than d_k_ with respect to at least one attribute a_i_ and is at least as good as d_k_ on all other attributes.*

Without loss of generality, we assume dominance by preferring a smaller value, so *d_j_* ≺ *d_k_* is equivalent to satisfying the following condition: ∀*a_i_* ∈ *A*, *d_j_*[*a_i_*] ≤ *d_k_*[*a_i_*] ∧∃ *a_h_* ∈ *A*, *d_j_*[*a_h_*] < *d_k_*[*a_h_*].

**Definition** **3.** **Snapshot skyline query**. *A snapshot skyline query over S retrieves those data objects in S_c_ that are not dominated by any other data object.*

Obviously, a snapshot skyline query over *S* is equivalent to a skyline query over *S_c_*. Therefore, we will simply refer to a snapshot skyline query as a skyline query for ease of presentation.

**Definition** **4.** **Continuous skyline query**. *For a given query time interval, the continuous skyline query is asked to sequentially compute the snapshot skyline query over S at each moment in the query time interval.*

**Figure 1:**
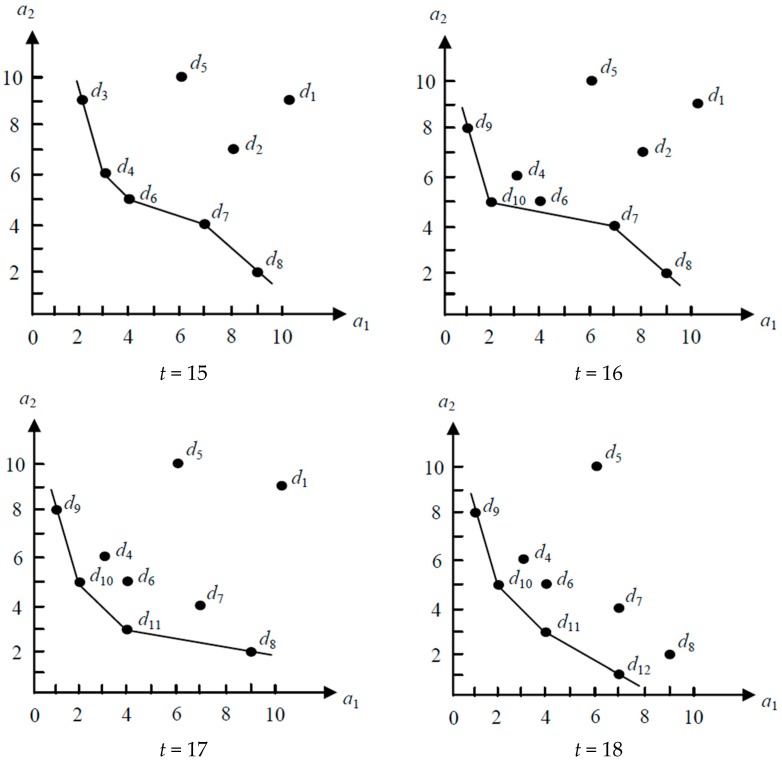
The example of a continuous skyline query being shown in transition from times 15 to 18.

Assume that Table 1 is the set of data objects perceived by sensor nodes in a WSN. Figure 1 shows an example, where the query results of a continuous skyline query over Table 1 is depicted in transition from time 15 to 18. We can see from Table 1 that each data object contains two attributes and has an arrival time and an expiration time associated with it. In Figure 1, those data objects connected by line segments are the query results of a continuous skyline query over Table 1. As shown in Figure 1, the query results of a continuous skyline query change dynamically over time. 

The key problem of a continuous skyline query is how to efficiently compute and incrementally update skyline query results in a given query time interval. In the following, we present a novel energy-efficient continuous skyline query method.

## 4. The Energy-Efficient Continuous Skyline Query Method 

In this section, we first depict the reference architecture of WSNs in Section 4.1 and then present a straightforward baseline solution to processing the continuous skyline query in Section 4.2. Lastly, in Section 4.3 we propose the energy-efficient continuous skyline query algorithm.

### 4.1. Reference Architecture of WSNs

**Figure 2:**
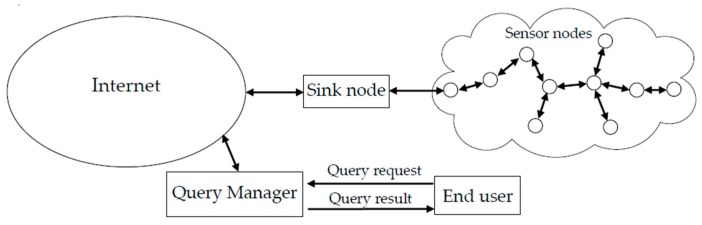
Reference architecture of a WSN.

Figure 2 illustrates the reference architecture of a WSN. As shown in Figure 2, a large number of distributed sensor nodes are organized into a multi-hop wireless network, which is connected to the Internet through a sink node. Moreover, a query manager is responsible for receiving, forwarding, and processing query requests from users for the perceptual data captured by distributed sensor nodes.

A user-initiated continuous skyline query request, denoted as *qr*, is represented as a 4-tuple, i.e., *qr* = <*csq*, *t_s_*, Δt, *t_e_* > where *csq* denotes that the user-initiated query is the continuous skyline query, *t_s_* is the start time of the query execution, Δt represents the time interval between two consecutive skyline queries, and *t_e_* denotes the end time of the continuous skyline query.

To process queries over the sensor data stream in a WSN, all sensor nodes in the WSN are organized into multiple clusters according to low-energy adaptive clustering hierarchy (LEACH) [23] in this paper. In LEACH, each cluster has one sensor node acting as the cluster head and all non-cluster head nodes transmit their data to the cluster head. The cluster head receives data from all the cluster members and transmits data to the sink node.

### 4.2. Baseline Approach

One of the simplest methods for processing continuous skyline query in a WSN is that all sensor nodes forward their valid data objects to sink node; sink node sends all the valid data objects to the query manager via the Internet; and then, the query manager computes skyline over all the valid data objects periodically using a centralized skyline query algorithm. We call the above method the centralized computing method for continuous skyline query in a WSN. Obviously, the centralized computing method asks all sensor nodes in a WSN to transmit their all valid data objects to sink node, which results in a large amount of energy consumption. Thus, it is not suitable for WSNs with limited energy. In addition, the method ignores the computing power of each sensor node itself and gives up on the in-network processing paradigm of WSNs in computing continuous skyline. 

In this subsection, we consider the in-network processing technique and propose a naive baseline approach (*BA*) for computing continuous skyline query in a WSN. Assume that sink node transmits the query request <*csq*, *t_s_*, Δt, *t_e_*> issued by a user to all cluster heads and each cluster head forwards <*csq*, *t_s_*, Δt, *t_e_* > to its cluster members. 

Specifically, *BA* includes the following steps.

(1) For the query request <*csq*, *t_s_*, Δt, *t_e_*>, each sensor node filters out its valid data objects according to the query execution time and the valid time interval [*t_arr_*(*d*), *t_exp_*(*d*)] of each data object *d*, employs a centralized skyline query algorithm such as BNL [8] or SFS [9] to compute the local skyline over all its valid data objects, and sends the local skyline query result to its cluster head.
(2) Each cluster head employs the centralized skyline query algorithm to compute the cluster’s skyline over the set of cluster’s candidate data objects and sends the query result to sink node. The set of cluster’s candidate data objects means the union of the local skyline query results computed by all sensor nodes located in the cluster.
(3) Sink node employs the centralized skyline query algorithm to compute the final skyline over the set of global candidate data objects and sends the final query result to the query manager. The set of global candidate data objects means the union of the cluster’s query results computed by all cluster heads.
(4) The query manager returns the query result to the user.
(5) When the next query execution time arrives, if it is less than or equal to *t_e_*, the execution returns to step (1). Otherwise, the query is terminated.

In this paper, we refer to the snapshot skyline query performed by each sensor node on its valid data objects as the local skyline query. The snapshot skyline query performed by each cluster head over the set of cluster’s candidate data objects is called the cluster’s skyline query. Similarly, we refer to the snapshot skyline query performed by sink node on the set of global candidate data objects as the final skyline query. 

Compared to the centralized computing method, *BA* reduces the overhead of data communication between sensor nodes by pre-computing the local skyline at each sensor node and the cluster’s skyline at each cluster head. However, the query results of the local skyline and the cluster’s skyline still contain some data objects that can be filtered out in advance, so further optimization and improvement are needed.

### 4.3. Energy-Efficient Continuous Skyline Query Algorithm

In this subsection, we present an energy-efficient continuous skyline query algorithm, called EECS. In EECS, a pruning strategy is proposed to reduce the overhead of data communication between sensor nodes. To efficiently prune the non-qualifying data objects for the final query result, we first define the concept of dominant capability for data objects. To facilitate the presentation, Table 2 summarizes the symbols we use throughout the following sections.

**Definition** **5.** **Dominant capability**. *For any data object d of S, the dominant capability of d, denoted by DC(d), is defined as follows: DC(d) =*$\prod_{i=1}^{m}\left(e_{i}-d\left[a_{i}\right]\right)$.

**Figure 3:**
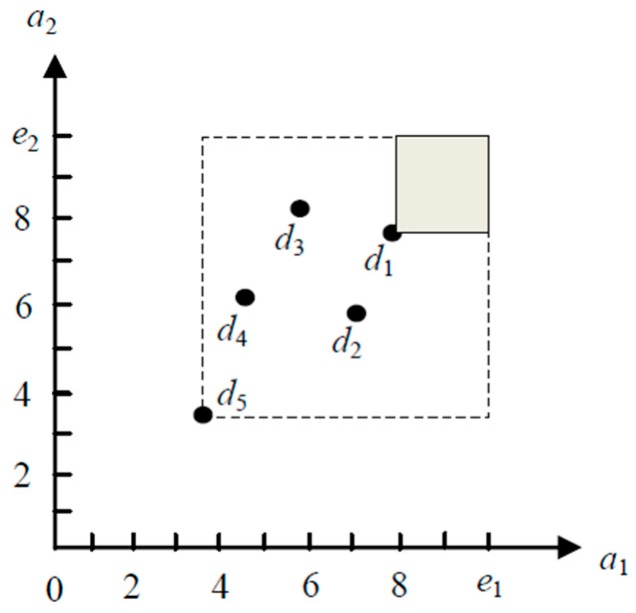
Illustration of dominant capability.

Figure 3 shows an example of dominant capability of two-dimensional data objects. In Figure 3, the area of the dotted rectangle represents the dominant capability of data object *d*_5_, and the data objects located in the area are dominated by *d*_5_. Similarly, the area of the filled small rectangle denotes the dominant capability of data object *d*_1_ in Figure 3, and the data objects located in the area are dominated by *d*_1_. Obviously, the dominant capability of a data object *d* reflects the dominance area of *d*, and all data objects falling within the dominance area must be dominated by *d*. The greater dominant capability means the larger dominance area.

Further, we give the definition of maximum dominance data object.

**Definition** **6.** **Maximum dominance data object**. *For a given set of valid data objects S_i_, we say*$d_{j}$ ($d_{j}\in S_{i}$*) is the maximum dominance data object of S_i_, if and only if* $\forall d_{k}\in S_{i}\left(DC\left(d_{k}\right)\leq DC\left(d_{j}\right)\right)$*. We use md(S_i_) to denote the maximum dominance data object of S_i_.*

Now, let us elaborate on EECS presented in this paper. EECS consists of 4 phases and its high-level description is as follows.

(1) *Preprocessing Phase*. In this phase, each sensor node (including cluster heads) deletes its expired data objects in real time, dynamically maintains its valid data objects, and employs a centralized skyline query algorithm to compute the local skyline over all its valid data objects. At the same time, each cluster head computes to get the maximum dominance data object of its local skyline query result.
(2) *Query Shipping Phase*. Sink node transmits the query request <*csq*, *t_s_*, Δt, *t_e_*> issued by a user to all cluster heads, and each cluster head forwards <*csq*, *t_s_*, Δt, *t_e_*> and its maximum dominance data object *md*(*S_i_*) to its cluster members.
(3) *Initial Skyline Calculation Phase*. This phase leverages the calculation results of preprocessing phase and efficient filtering strategy to obtain the skyline query result at the initial query time *t_s_* by means of in-network computation.
(4) *Incremental Update Phase*. The phase incrementally updates the calculation result of the previous skyline query to compute the query results at the subsequent continuous query time, which avoids the extra computational overhead and data communication cost caused by continuous repetitive skyline calculations.

The procedure of the *Initial Skyline Calculation Phase* is described as follows.

(1) *Node Processing*. Each sensor node uses the received *md*(*S_i_*) to filter out the non-qualified data objects of its local skyline query result generated at *t_s_* and get the filtered local skyline query result. Here, the non-qualified data objects refer to those data objects of the local skyline query result dominated by *md*(*S_i_*). At the same time, each sensor node sends the filtered local skyline query result to its cluster head and keeps a copy of the filtered local skyline query result locally.
(2) *Cluster Head Processing*. Each cluster head merges its filtered local skyline query result with all received filtered local skyline query results from its cluster members to get its collection of cluster’s candidate data objects, employs the centralized skyline query algorithm to compute its cluster’s skyline query, sends the query result to sink node, and keeps a copy of the query result locally.
(3) *Sink Node Processing*. Sink node merges all received query results from cluster heads to get its collection of global candidate data objects, employs the centralized skyline query algorithm to compute the final skyline over the collection of global candidate data objects, sends the final query result to the query manager, and keeps a copy of the final result locally. At the same time, the query manager returns the query result to the user.

The procedure of the *Incremental Update Phase* is described as follows.

(1) When the next query time arrives, if it is greater than *t_e_*, sink node terminates the continuous skyline query. Otherwise, the following steps are executed.
(2) Sink node deletes all expired data objects in its copy of the final result at the new arrival query time. Then, sink node computes to get the maximum dominance data object *md*(*S_f_*) of its copy of the final result *S_f_* and sends *md*(*S_f_*) to all cluster heads.
(3) Each cluster head modifies its copy of the query result by deleting all expired data objects and those data objects dominated by *md*(*S_f_*), computes to get the maximum dominance data object *md*(*S_c_*) of its copy of the query result *S_c_*, and sends *md*(*S_c_*) and *md*(*S_f_*) to its all cluster members.
(4) Each sensor node modifies its copy of local skyline query result by deleting all expired data objects of the copy and then does a dominant check for each new valid data object that has been added since the last query. Specifically, the method of dominant check is as follows: For a new added valid data object *d_k_* in a sensor node, if *d_k_* is dominated by any data object of the copy of local skyline query result or one of *md*(*S_c_*) and *md*(*S_f_*), *d_k_* is filtered out; otherwise, *d_k_* is added into a local candidate list, and all data objects in the copy of local skyline query result dominated by *d_k_*, if exist, are deleted. Finally, each sensor node sends all data objects of its local candidate list to its cluster head and adds all data objects of its local candidate list into its copy of local skyline query result.
(5) Each cluster head does a dominant check for each data object received from these local candidate lists of its cluster members. Specifically, for each data object *d_i_* received from cluster members, if *d_i_* is dominated by any data object of the copy of the query result, *d_i_* is filtered out; otherwise, *d_i_* is added into a cluster candidate list, and all data objects in the copy of the query result dominated by *d_i_*, if exist, are deleted. Then, each cluster head sends all data objects of its cluster candidate list to sink node and adds all data objects of its cluster candidate list into its copy of the query result.
(6) Sink node does a dominant check for each data object received from cluster heads. Specifically, for each data object *d_j_* received from cluster heads, if *d_j_* is dominated by any data object of the copy of the final result, *d_j_* is filtered out; otherwise, *d_j_* is added into the copy of the final result, and all data objects in the copy of the final result dominated by *d_j_*, if exist, are deleted. Then, sink node sends all data objects of the copy of final query result to the query manager, and the query manager returns them to the user.

Compared with *BA*, EECS has two obvious advantages. First, EECS adopts the filtering strategy based on maximum dominance data object to significantly reduce communication costs in the continuous skyline query; secondly, EECS proposes the incremental update strategy that uses the result of the previous skyline query to incrementally generate the next skyline query result with little computation and communication overhead.

## 5. Performance Evaluation

In this section, we evaluate the performance of the proposed method (EECS) with *BA*, MINMAX and SWSMA through extensive experiments. In what follows, we will first describe the experimental settings and then present the simulation results. 

### 5.1. Experimental Setting

Experiments are conducted on a PC with a 3.2 GHz Intel CPU and 4G Byte of memory, running Win7 with 32 bit. The simulated sensor network is implemented using MATLAB. The simulations used in our experiments can adjust parameters such as the number of sensor nodes, the transmission range, and the size of the network area. The default network area is a rectangle grid of 200 m × 200 m, where 100–500 sensor nodes are randomly distributed. The sink node is placed at the center of the network. The communication range of each sensor node varies from 20m to 30m. For EECS and the reference baseline methods (*BA* and MINMAX), sensor nodes are organized according to the following two structures: (1) multiple clusters based on LEACH [23] for EECS and *BA*, and (2) routing tree based on the shortest path for MINMAX and SWSMA. 

For performance evaluation, we adopt the synthetic datasets including the two data distribution, independent and anti-correlated, which are commonly used for skyline query. The dimension of data objects ranges from 2 to 5, and the cardinality of dataset is in the range of 10k to 20k. For each data object, an arrival time and an expiration time associated with it are assigned. Specifically, for each data object, we randomly pick an arrival time between *t_s_* − 10 and *t_e_* + 10, where *t_s_* is the start time of the continuous skyline query, and *t_e_* denotes the end time of the continuous skyline query. Then, we pick the expiration time randomly between the arrival time and *t_e_* + 10. In our simulation experiments, each data object will arrive and be deleted according to its arrival time and expiration time, and the skyline query is continuously computed. The size of query time interval [*t_s_*, *t_e_*] for the continuous skyline query varies from 200 to 400. Similar to the literature [20], we choose *total communication cost* as the main performance metric. *Total communication cost*, called TCC, denotes the number of the messages transmitted for computing the continuous skyline query in a time interval [*t_s_*, *t_e_*]. Table 3 summarizes the main parameters and their settings.

### 5.2. Experimental Results

**Figure 4:**
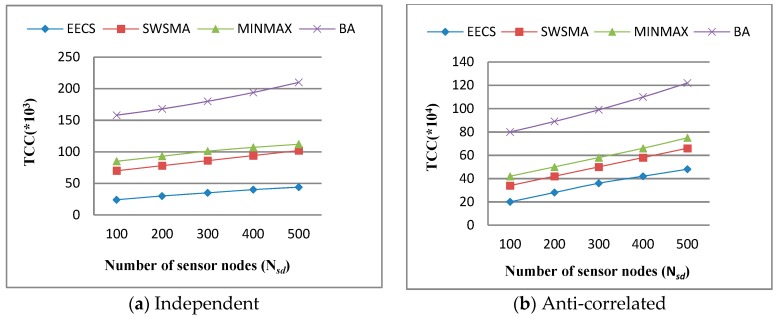
Performance vs. number of sensor nodes.

We first evaluate the influence on performance by the number of sensor nodes. Figure 4 depicts *total communication cost* (TCC) as a function of the number of sensor nodes when other simulation parameters are set to their default values. We can see from Figure 4 that TCC of each method increases accordingly with the number of sensor nodes over the independent dataset and the anti-correlated dataset. This is not surprising because the increase in the number of sensor nodes leads to the corresponding increase in communication overhead between sensor nodes. We can also see from Figure 4 that EECS significantly outperforms the other three methods in terms of TCC. The reason is that EECS reduces the overhead of data transmission between sensor nodes through efficient incremental update and filtering strategy.

**Figure 5:**
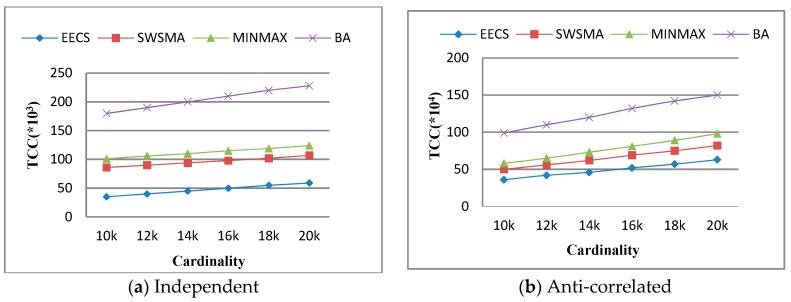
Performance vs. cardinality.

Then, we study the effect of cardinality on *total communication cost* (TCC). Figure 5 illustrates that TCC as a function of the cardinality of dataset over independent dataset and anti-correlated dataset when other simulation parameters are set to their default values. The results show that TCC of each method increases accordingly with the cardinality of dataset. This is because the increase in the cardinality of dataset leads to the corresponding increase in the amount of data objects processed and transmitted by sensor nodes. We can see from Figure 5 that EECS is obviously better than the other three methods in terms of TCC. The reason is EECS is optimized for filtering strategy and skyline incremental maintenance. 

**Figure 6:**
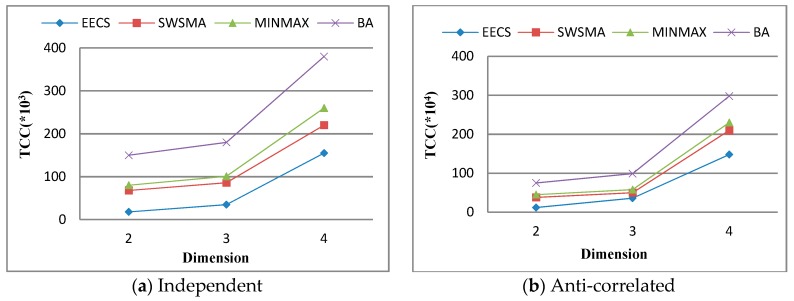
Performance vs. dimension.

Further, we study the effect of data dimension on *total communication cost* (TCC). Figure 6 plots that TCC as a function of dimension over independent dataset and anti-correlated dataset when other simulation parameters are set to their default values. We can see from Figure 6 that TCC of each method increases accordingly with data dimension. This is not surprising because the increase in dimension usually leads to the corresponding increase in the number of data objects contained a skyline query result set, which inevitably leads to an increase in the amount of data objects transmitted between sensor nodes. We can also see from Figure 6 that EECS significantly outperforms the other three methods in terms of TCC. The reason is the same as the one for Figure 5.

**Figure 7:**
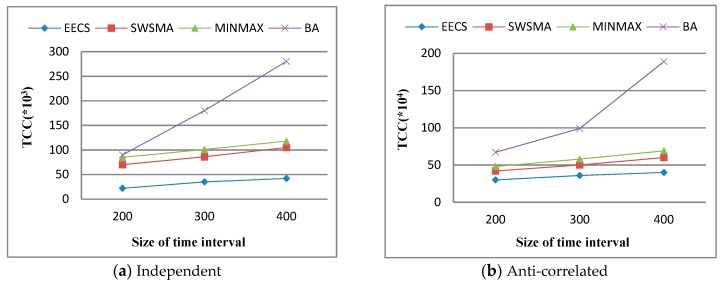
Performance vs. size of query time interval.

Finally, we study the effect of query time interval [*t_s_*, *t_e_*] on *total communication cost* (TCC). Figure 7 depicts TCC as a function of query time interval over independent dataset and anti-correlated dataset when other simulation parameters are set to their default values. The results show that TCC of each method increases accordingly with the size of query time interval. This is because the increase in the size of query time interval leads to an increase in the number of skyline queries. We can see from Figure 7 that EECS significantly outperforms the other three methods in terms of TCC. The reason is the same as the one for Figure 5.

## 6. Conclusions

This paper addresses the problem of continuous skyline query processing in WSNs. The continuous skyline query is to continuously compute a skyline query over a multidimensional dataset in which each data object has an arrival time and an expiration time associated with it. Most sensor nodes in a WSN are battery powered, and wireless communication is one of the major consumers of the sensor energy. Thus, the challenge for the continuous skyline query in WSNs is to fulfill the query task with a minimum communication cost. In this paper, we present an energy-efficient continuous skyline query method, called EECS. It aims at reducing communication cost on processing continuous skyline query in WSNs. First, the concept of maximum dominance data object is defined for pruning strategy. Then, a continuous skyline query processing in WSNs is finely abstracted into four hierarchical phases. Finally, the optimization process for each phase significantly reduces the overhead of data transmission between sensor nodes by means of real time preprocessing, in-network skyline computation, skyline incremental update, and pruning strategy. Extensive experiments are conducted, and the experimental results demonstrate the effectiveness of our methods. 

## Figures and Tables

**Table 1:** The set of data objects with arrival and expiration times.

*id*	*a*_1_	*a*_2_	*t_arr_*	*t_exp_*
*d*_1_	10	9	1	17
*d*_2_	8	7	2	16
*d*_3_	2	9	4	15
*d*_4_	3	6	6	18
*d*_5_	6	10	7	22
*d*_6_	4	5	9	23
*d*_7_	7	4	11	21
*d*_8_	9	2	13	24
*d*_9_	1	8	16	26
*d*_10_	2	5	16	28
*d*_11_	4	3	17	27
*d*_12_	7	1	18	30

**Table 2:** Symbols used in this paper.

Symbols	Descriptions
*S*	The set of *m*-dimensional data objects perceived by sensor nodes in a sensor network
{*a*_1_, *a*_2_, …, *a_m_*}	The set of attributes of each data object in *S*
*d*	A data object of *S*
*d*[*a_i_*]	The *i*-th attribute value of *d*
[*b_i_*, *e_i_*]	The domain on attribute *a_i_*, i.e., for any data object *d*, *b_i_* ≤ *d*[*a_i_*] ≤ *e_i_*
*DC*(*d*)	The dominant capability of *d*
*md*(*S_i_*)	The maximum dominance data object of data object set *S_i_*

**Table 3:** Main simulation parameters.

Parameter	Range	Default Value	Description
N*_sd_*	100–500	300	Number of sensor nodes
Cardinality	10–20k	10k	Number of data objects
Dimension	2–4	3	Number of attributes each data object contains
[*t_s_*, *t_e_*]	[0, 200], [0, 300], [0, 400]	[0, 300]	Query time interval
